# The Effect of Vitamin D Supplements on Clinical and Para-Clinical Outcomes in Patients With Multiple Sclerosis: Protocol for a Systematic Review

**DOI:** 10.2196/12045

**Published:** 2019-04-22

**Authors:** Mahsa Ghajarzadeh, Abbas Ali Keshtkar, Amirreza Azimi, Mohammad Ali Sahraian, Mehdi Mohammadifar, Sreeram V Ramagopalan

**Affiliations:** 1 Universal Council of Epidemiology Universal Scientific Education and Research Network Tehran University of Medical Sciences Tehran Islamic Republic of Iran; 2 Department of Health Sciences Education Development School of Public Health Tehran University of Medical Sciences Tehran Islamic Republic of Iran; 3 MS Research Center Neuroscience Institute Tehran University of Medical Sciences Tehran Islamic Republic of Iran; 4 Department of Radiology Zanjan University of Medical Sciences Zanjan Islamic Republic of Iran; 5 Bristol-Myers Squibb Uxbridge United Kingdom

**Keywords:** multiple sclerosis, systematic review, relapse, magnetic resonance imaging, chemokines

## Abstract

**Background:**

Multiple sclerosis (MS) is an inflammatory disease, which has a wide range of effects on patients. There are controversies regarding the role of vitamin D in clinical and laboratory improvements in MS patients.

**Objective:**

The aim of this systematic review protocol is to evaluate the efficacy of vitamin D supplements on relapse rate, gadolinium-enhancing lesions of magnetic resonance imaging (MRI), and cytokine profiles.

**Methods:**

We will search PubMed, Scopus, EMBASE, CINAHL, Web of Science, Ovid, ProQuest, American College of Physicians Journal Club database, Health Technology Assessment Database (The Cochrane Collaboration), and National Health System Economic Evaluation Database (The Cochrane Collaboration) and gray literature including reference of included studies and conference abstracts. Clinical trials reporting the effect of any doses of vitamin D on relapse rate, gadolinium-enhancing lesions of MRI, and cytokine profiles will be included. In total, 2 independent researchers will independently assess the studies, extract data, and evaluate the quality of primary studies.

**Results:**

This systematic review was started in September 2017 and the process is continuing. The included articles are evaluated and researchers are going to extract the data.

**Conclusions:**

To our knowledge, this will be the first comprehensive systematic review aiming to assess the effect of vitamin D supplements on clinical and para-clinical outcomes in patients with multiple sclerosis.

**International Registered Report Identifier (IRRID):**

DERR1-10.2196/12045

## Introduction

### Background

Multiple sclerosis (MS) is an inflammatory progressive disorder of the central nervous system, which affects women more than men [1]. The annual incidence of the disease has increased, and environmental and genetic factors are considered important in disease development [2-4].

Smoking, Epstein-Barr virus infection, habitat latitude, and vitamin D status are among the environmental factors associated with disease risk [5,6].

Previous studies have demonstrated that a longer duration of sunlight exposure, decreasing latitude of residence, and a vitamin-rich diet are associated with a lower risk of developing MS [7-9].

Vitamin D, which is a modulator of calcium and phosphorus, plays an important role in bone formation and maintenance as well as having anti-inflammatory, antiproliferation, and immune intonation functions [10]. The regulation of gene expression in immune cells is thought to explain vitamin D’s immune effects on cells [11].

Experimental studies have shown that vitamin D administration could slow down experimental autoimmune encephalomyelitis progression [12,13]. In humans, lower vitamin D status has been reported at the time of relapse in MS patients as compared with the relapse-free period [14-16]. Some previous studies showed that vitamin D supplements were not effective in reducing MS-related relapses [17,18], whereas others revealed that vitamin D supplements were helpful in reducing relapse rates and MRI lesions in MS patients [19,20].

A systematic review and meta-analysis conducted previously showed that vitamin D supplementation was not beneficial to control MS relapses (odds ratio 0.98, 95% CI 0.45-2.16) by including 5 randomized clinical trials (RCTs) [21].

The main goal of this systematic review and meta-analysis will be to evaluate the effectiveness of vitamin D supplementation on clinical and para-clinical outcomes in patients with multiple sclerosis

### Objectives

#### Primary Objective

The primary objective was to identify the efficacy of vitamin D supplement administration in patients with MS on relapse rates.

#### Secondary Objectives

The secondary objectives were the following:

1. Identify the efficacy of vitamin D supplement administration in patients with MS on gadolinium-enhancing lesions.
2. Identify the efficacy of vitamin D supplement administration in patients with MS on cytokine profiles.
3. Identify the efficacy of vitamin D supplement administration in patients with MS on disability.

## Methods

### Study Characteristics

We will include RCTs, being single-blinded or double-blinded or open-label trials in which relapse rate was one of the main outcomes after vitamin D supplement therapy. The articles that had been published in the English language will be included.

Cohort studies, case-control studies, and any other types of studies will be excluded.

### Types of Participants

We will include studies with adult participants (aged more than 18 years) with the relapsing-remitting (RR) form of the disease (as we want to assess the effect of the intervention on relapse rate).

### Control Group

The control group should be patients who received placebo (color, shape, and smell similarity with the vitamin D supplements).

### Types of Intervention

We considered interventions as vitamin D supplements (any doses) whereas controls should have received placebos.

### Outcome Assessment

The primary outcome assessment will be carried out by a report of relapse, which is characterized by attacks of new or increasing neurologic symptoms (such as double vision, blurred vision, numbness, the lack of balance, memory loss, muscle cramping secondary to spasticity, bladder, bowel, and sexual dysfunction, bilateral facial weakness or trigeminal neuralgia, nystagmus, and intention tremor, and heat intolerance). The secondary outcomes will be assessed by comparing the number of gadolinium-enhanced plaques before and after the treatment, level of cytokines (tumor necrosis factor and interleukins) before and after the treatment, and progression of disability measured with the Expanded Disability Status Scale (EDSS).

### Information Sources

We will search PubMed, Scopus, EMBASE, CINAHL, Web of Science, Ovid, ProQuest, American College of Physicians Journal Club database, Health Technology

Assessment Database (The Cochrane Collaboration), and National Health System Economic Evaluation Database (The Cochrane Collaboration) and gray literature including reference of included studies and conference abstracts.

### Search Strategy

A search strategy was developed, and it will be used to search in all databases. The search keywords are as following:

1. Multiple sclerosis
2. MS
3. Relapsing-Remitting
4. RR
5. Multiple Sclerosis, Relapsing-Remitting
6. Remitting-Relapsing Multiple Sclerosis
7. Multiple Sclerosis, Remitting-Relapsing
8. Remitting Relapsing Multiple Sclerosis
9. Relapsing-Remitting Multiple Sclerosis
10. Relapsing -Remitting Multiple Sclerosis
11. Multiple Sclerosis, Acute Relapsing
12. Acute Relapsing Multiple Sclerosis
13. Vitamin D
14. 25 Hydroxyvitamin D 2
15. 25-Hydroxyergocalciferol
16. 25 Hydroxyergocalciferol
17. 25-Hydroxyvitamin D2
18. 25 Hydroxyvitamin D2
19. Ercalcidiol
20. 25-Hydroxycalciferol
21. 25 Hydroxycalciferol
22. Dietary Supplement
23. Supplements, Dietary
24. Dietary Supplementations
25. Supplementations, Dietary
26. 1 or 2
27. 3 or 4
28. 5 or 6 or 7 or 8 or 9 or 10 or 11 or 12
29. 13 or 14 or 15 or 16 or 17 or 18 or 19 or 20 or 21
30. 22 or 23 or 24 or 25
31. 26 and 27 and 28 and 29 and 30

### Study Records

In total, 2 independent researchers will independently assess the articles. All obtained articles will be screened by title and abstract, and selected articles will be considered as eligible, not eligible, or may be eligible.

Articles that are considered as not eligible by both researchers will be excluded and others will be searched for by obtaining the full text. Eligible and may be eligible articles will be assessed independently, and in the case of a disagreement, a session for solving the disagreement will be held. The researchers will extract the data from papers and in the case of incomplete/unclear information, surveys will be sent to the authors.

### Data Items

Data regarding the name of authors, year of publication, number of patients, journal title, date, demographic data, method of intervention, dose of vitamin D supplement, duration of the study, frequency of relapses during the study, mean vitamin D levels at baseline and at the end of the study, type of control, number of baseline and final gadolinium enhanced plaques in MRI, and mean levels of cytokines and EDSS at baseline and the end of the study will be extracted by 2 independent researchers and will be recorded.

### Assessment of Risk of Bias in Included Studies

On the basis of the Cochrane Collaboration Risk of Bias assessment tool, 2 researchers will assess the risk of bias in each study [22]. Thus, each article will be categorized into low risk, high risk, and unclear risk. In the case of a disagreement, a discussion to reach an agreement will be held.

### Data Synthesis

When adequate studies are included, the meta-analysis will be performed by considering the relapse, the number of gadolinium-enhanced plaques, and the level of cytokines and disability (which is assessed by means of EDSS) as the main outcome. For all of the main outcomes, mean differences and standardized mean differences will be applied. In the case of missing SD or SE, it will be calculated directly using the data where possible. If adequate studies are retrieved, a subgroup analysis will be carried out.

### Subgroup Analysis

A subgroup analysis will be carried out according to sex, age, and EDSS.

## Results

This systematic review was started in September 2017, and the search process is completed. The articles are retrieved and are under review by 2 independent researchers. The findings of this systematic review will determine the effectiveness of vitamin D supplements on the prevention of MS relapses, MRI findings, and cytokines. The results of this systematic review will be published in a peer-reviewed journal.

## Discussion

MS is a disabling disease and nearly 85% of patients present with the RR form [23]. Relapses are not predictable and have negative impacts on a patient’s quality of life [24].

Since 1970, vitamin D has been considered as an important factor in MS development. Studies showed that the prevalence of MS is higher in regions with less exposure to ultraviolet light [25]. Other studies demonstrated that higher levels of serum vitamin D levels are associated with less disease activity in patients and by each doubling of serum level of this vitamin, the risk of relapse decreased by 27% [16]. Vitamin D supplements were considered to be useful for the reduction of disease-related MRI lesions, disability, and reduced relapse rates during the study period [17], but there is no consistent finding on the effectiveness of vitamin D supplements in all those studies. This may be because different studies administered different doses of supplements and they assessed the effects on different outcomes.

The results of this systematic review could be helpful for clinicians to understand the best dose of vitamin D supplement for patients with MS.

## Abbreviations

EDSS: Expanded Disability Status Scale
MRI: magnetic resonance imaging
MS: multiple sclerosis
RCT: randomized clinical trial
RR: relapsing-remitting
